# Supplementary material for: Dynamical-invariant-based holonomic quantum gates: Theory and experiment
Source: Fundam Res. 2022 Feb 17;3(2):229–36. doi: 10.1016/j.fmre.2021.11.036 (PMC11630703; doi:10.1016/j.fmre.2021.11.036)
Supplement: Supplementary Data S1 — Supplementary Raw Research Data. This is open data under the CC BY license http://creativecommons.org/licenses/by/4.0/ [file mmc1.pdf]

Reply to the referees' report

Reviewer 1: In this manuscript, the authors claim that they report a systematic, scalable approach to HQGs without using additional Hilbert spaces based on dynamical invariant. Their motivation is to improve the existing non-adiabatic HQGs, which requires additional Hilbert spaces. From their manuscript, it seems that their scheme is performed by using abelian geometric phases, i.e., Aharonov-Anandan phases (seen from PRL 58, 1593 (1987)). As a matter of fact, the abelian-geometric-phase-based quantum computation has already been proposed by Wang (PRL 87, 097901) and Zhu (PRL 89, 097902) in 2001 and 2002. The two schemes only use the qubit space itself, i.e., they do not need the additional Hilbert space. From this point, this manuscript does not represent an advance compared with the previous schemes, such as the schemes of Wang and Zhu. The only difference is that this manuscript utilizes the dynamical invariant theory to solve the Schrodinger equation. The advantage of this approach is not be well presented, at least from the basic theory.

Our response: We appreciate the Referees for spending their precious time on reviewing our manuscript! We would like to defend that our result is a large step forward compared with other proposals of HQGs. We aim to propose a systematic method of implementing scalable and platform-independent HQGs, not only to propose a auxiliary-state-free non-adiabatic HQG. We believe that our manuscript is suitable for publication in fundamental research for the following reasons.

First, our design of HQGs is systematic. We introduced a concrete process of finding a certain HQG in our manuscript. We use the analytic solution to the Schrödinger equation of the n-qubit Hamiltonian. Given the analytic solution, we only need to find the parameters that perform an n-qubit operation, and finding the parameters is program-solvable. Such a process takes all the coupling terms in the Hamiltonian at the same time; hence, we will be able to entangle multiple qubits within a few pulses, or perform single-qubit operations on a large scale quantum computing system without extra concern of decoupling.

Second, the design of HQGs in our protocol is scalable. An elementary requirement for large-scale quantum computers is that the design of error-invulnerable gates should be scalable. Previous DI-based HQGs has the problem of solving the DI equation beyond two-level systems, and hence are not able to design higher-qubit quantum gates. In fact, no other DI-based HQGs have two-qubit gates yet. Moreover, other HQG protocols cannot scale up easily either. For example, the well-known HQG protocol in terms of parametric driving suffers the scalability problem, because parametrizing the higher-qubit Hamiltonian not only involves integrating special functions such as the Bessel function, but also requires different techniques of parametrizing the subspace of the system when designing different quantum gates. Overall, previous HQG protocols, whether DI-based or not, confront tricky situations when dealing with higher number of qubits. Our protocol, on the other hand, is able to perform n-qubit HQGs. As an example, we designed HQGs on a three-qubit spin-chain system and a four-qubit spin-chain system in our manuscript.

Third, our HQG protocol is platform-independent. Most of quantum computing systems have Hamiltonians that consist of single-qubit control terms, single-qubit Zeeman terms, and Ising-like, XX-like or XXY-like coupling terms. Other protocols of HQGs are usually proposed for specific systems because for distinct Hamiltonians the stories are completely different to design evolution passages in the Hilbert space, rendering these protocols are usually platform-dependent. Nevertheless, in the Lie-algebraic point of view, the apparently distinct Hamiltonians are of the same type. Therefore, our method not only works for two-level systems but also fits well for systems of higher-spins, as long as we use the $\mathrm{su}(n)$ generators to expand the Hamiltonian. For example, a typical three-level XX-coupling Hamiltonian that is commonly used in superconducting circuit reads

$$\begin{aligned}\mathcal{H} =&\Delta_1|1\rangle\langle 1|\otimes I+\Delta_2 I\otimes|1\rangle\langle 1|+(2\Delta_1+\eta)|2\rangle\langle 2|\otimes I+(2\Delta_2+\eta)I\otimes|2\rangle\langle 2|\\ &+g(\sigma^+\otimes\sigma^-+\sigma^-\otimes\sigma^+)+\frac{\Omega_1}{2}(\mathrm{e}^{\mathrm{i}\omega t}\sigma^-+\mathrm{e}^{-\mathrm{i}\omega t}\sigma^+)\otimes I+\frac{\Omega_2}{2}I\otimes(\mathrm{e}^{\mathrm{i}\omega t}\sigma^-+\mathrm{e}^{-\mathrm{i}\omega t}\sigma^+),\end{aligned} \tag{1}$$

where σ^+ and σ^- are the creation and annihilation operators. We can rewrite this Hamiltonian in terms of the Gell-Mann matrices

$$\begin{aligned}\mathcal{H} =&\frac{\Delta_1}{6}(\sqrt{3}\lambda_8-3\lambda_3)\otimes I+\frac{\Delta_2}{6}I\otimes(\sqrt{3}\lambda_8-3\lambda_3)-\frac{(2\Delta_1+\eta)}{\sqrt{3}}\lambda_8\otimes I-\frac{(2\Delta_2+\eta)}{\sqrt{3}}I\otimes\lambda_8\\ &+\frac{g}{2}(a\otimes a+b\otimes b)\\ &+\frac{\Omega_1}{2}\cos(\omega t+\phi_1)a\otimes I-\frac{\Omega_1}{2}\sin(\omega t+\phi_1)b\otimes I+\frac{\Omega_2}{2}\cos(\omega t+\phi_2)I\otimes a-\frac{\Omega_2}{2}\sin(\omega t+\phi_2)I\otimes b,\end{aligned} \tag{2}$$

where $a=\lambda_1+\sqrt{2}\lambda_6$ and $b=\lambda_2+\sqrt{2}\lambda_7$. According to our Lie-algebraic argument, the DI for the Hamiltonian (2) is

$$\begin{aligned}\mathcal{I} =&(\omega-\Delta_1)\lambda_3\otimes I+(3\omega-3\Delta_1-2\eta)\lambda_8\otimes I+(\omega-\Delta_2)I\otimes\lambda_3+(3\omega-3\Delta_2-2\eta)I\otimes\lambda_8\\ &+g(a\otimes a+b\otimes b)\\ &+\Omega_1\cos(\omega t+\phi_1)a\otimes I-\Omega_1\sin(\omega t+\phi_1)b\otimes I+\Omega_2\cos(\omega t+\phi_2)I\otimes a-\Omega_2\sin(\omega t+\phi_2)I\otimes b.\end{aligned} \tag{3}$$

Designing HQGs in a three-level system is then possible, no matter if we use the two-dimensional subspaces of the three-level system as qubits or regard the three-level system as qutrits. Therefore, our method works for various quantum computing systems including nuclear magnetic resonance, superconducting circuits and nitrogen-vacancy centers.

The Abelian-geometric-phase-based quantum gates proposed by Wang and Zhu in 2001 and 2002 is neither systematic nor scalable. They can only implement a limited number of two-qubit gates, let alone higher-qubit HQGs; hence, using their protocol will greatly complicate the quantum circuit. Moreover, cancelling the dynamical phase in their scheme requires a second loop, which will double the gate length and further lengthen the circuit. Relying on a second loop also makes their geometric gates hard to optimize. On the contrary, our protocol of designing HQGs permits multiple solutions, which may suit for situations requiring different gate lengths, as discussed in our manuscript.

In conclusion, our DI-based HQGs are more systematic, scalable and platform-independent than other HQG protocols. In the upcoming NISQ quantum computing era, these three criteria are of great importance for developing robust quantum gates, so our work meets the high-standard fundamental research requirement in novelty.
